# Influence of Parental Exposure to Risk Factors in the Occurrence of Oral Clefts

**DOI:** 10.30476/DENTJODS.2019.77620.0

**Published:** 2020-06

**Authors:** Sandra Regina Altoé, Álvaro Henrique Borges, Ana Thereza de Saboia Campos Neves, Andreza Maria Fábio Aranha, Alexandre Meireles Borba, Mariano Martinez Espinosa, Luiz Evaristo Ricci Volpato

**Affiliations:** 1 Dept. of Public Health, Nursery School, University of Cuiabá, Cuiabá, Brazil; 2 Dept. of Endodontics, Dental School, University of Cuiabá, Cuiabá, Brazil; 3 Dept. of Pediatric Dentistry, Dental School, University of Cuiabá, Cuiabá, Brazil; 4 Dept. of Surgery, Dental School, University of Cuiabá, Cuiabá, Brazil; 5 Dept. of Statistics, Federal University of Mato Grosso, Cuiabá, Brazil

**Keywords:** Case-Control Studies, Cleft Lip, Cleft Palate, Risk Factors

## Abstract

**Statement of the Problem:**

Non-syndromic cleft lip and palate are the most frequent craniofacial abnormalities in humans. The genetic, environmental and behavioral factors involved in this malformation must be clarified in different parts of the globe in the view of implementing preventive measures.

**Purpose:**

To analyze the influence of parental exposure to risk factors on the occurrence of oral clefts.

**Materials and Method:**

A case-control study was conducted with 150 mothers of oral cleft children paired by the children’s gender to 300 mothers of children without congenital anomalies from Mato Grosso, Brazil, for the study of the variables: gender and race/color of the children; parental educational level; age; number of pregnancies; prenatal care; obesity; stress; diabetes; hypertension; use of medications, alcohol and illicit drugs; smoking and exposure to ionizing radiation during the first trimester of pregnancy. The results were analyzed in relation to the chances possibility of each variable for the occurrence of oral cleft through the bivariate and multivariate analysis by applying the model of logistic regression.

**Results:**

Passive smoking, obesity, exposure to ionizing radiation and use of antibiotics were associated with the presence of clefts. The use of folic acid and analgesics were identified as preventive factors. The father's low educational level was found as a risk factor, while the black race/color was a preventive factor; nevertheless these variables were not associated in the multivariate analysis.

**Conclusion:**

The results reinforce the need to follow up the pregnant women, especially in the first trimester of pregnancy, in order to control the identified risk factors. Considering the factors associated with occurrence of oral clefts and those associated with its prevention, it is possible to apply specific health promotion measures during pregnancy, which can result in the reduction of oral clefts’ occurrence.

## Introduction

Non-syndromic cleft lip and palate are the most frequent craniofacial abnormalities in humans [ 1 ]. Its occurrence is highly variable according to gender, race, geographical location, environmental exposures and socioeconomic condition [ 1 - 3 ]. Its prevalence worldwide is approximately of 1.2/1000 live births [ 1 ] and in Brazil it varies from 0.19 to 1.54/1000 live births [ 3 ], leading to an estimated incidence of approximately 4,000 new cases per year, representing a significant public health issue [ 4 ]. Maternal exposure to risk factors in the first trimester of pregnancy has been associated with the occurrence of orofacial clefts because of the interference caused in the fusion of the craniofacial processes that form the primary and secondary palates, involving the lip, alveolar process, hard palate and soft palate, between the fourth and the twelfth week of pregnancy. As between the 6th and the 8th week of gestation the fusion of the upper lip is completed and between the 8th and 12th week the hard and soft palate’s fusion are completed, interferences in this period may lead to cleft lip, cleft palate and cleft lip and palate [ 5 ]. Aesthetic and functional problems caused by oral clefts require long-term treatment. They represent a higher risk of morbidity; difficulty in feeding, changes in speech and hearing affect the social interaction of the individual and cause psychological and financial impact in their families [ 1 - 5 ].

Studies aiming to understand the interaction between genetic, environmental and behavioral factors involved in this malformation in early pregnancy have been made in the view of implementing preventive measures [ 3 - 4 ]. Among the environmental and behavioral factors associated some relevant ones are smoking [ 5 - 7 ], alcohol consumption [ 5 , 8 - 9 ], birth order, birth interval [ 10 ], folic acid deficiency [ 11 - 12 ], parental age [ 8 , 11 - 12 ], race/color of skin [ 13 - 15 ], diabetes, hypertension [ 16 - 18 ], use of medications [ 8 , 19 ] and exposure to ionizing radiation [ 20 - 21 ]. It is possible to intervene in these factors with health promotion measures particularly during pregnancy, which can result in reduction of oral clefts’ occurrence.

This study aims to analyze the influence of parental exposure to risk factors on the occurrence of oral clefts; a priority for the advancement of research on the field [ 4 ].

## Materials and Method

A case-control study was conducted with genitors of the State of Mato Grosso, Mid-West Brazil. A total of 878 medical records of patients undergoing treatment at the Oral Clefts Rehabilitation Service of the General Hospital of the University of Cuiabá were analyzed for the composition of the case group. Among them, the mothers of children were selected according to the following inclusion criteria: presenting non-syndromic isolated cleft lip and/or palate; being under six years old, and the pregnancy period occurred in the State of Mato Grosso. From the 200 progenitors of children who met the inclusion criteria, 150 attended the hospital on the dates scheduled to participate in the study. The control group, in the proportion of two controls for each case, was paired according to the gender of the children in the case group, totalizing 300 genitors in the group. The genitors from the control group were enrolled in the University General Hospital (n= 138) and Santa Helena Hospital (n= 212). Both institutions constitute the State Hospital Reference System for the High Risk Pregnancy Assistance in Cuiabá. The inclusion criterion was the woman be mother of a newborn without malformations detected at birth and the exclusion criterion was the woman had spent the pregnancy period outside Mato Grosso. Therefore, the entire study population consisted of 450 genitors.

A structured questionnaire was used for data collection. It had questions about the child, the parents and maternal exposure to risk factors in the first trimester of pregnancy. The questionnaire was administered to the genitors of the study group from March 2012 to September 2014. The data collection from the control group occurred from May to September 2014. Before using the questionnaires, formal consent was obtained from the executive board of both hospitals; Ethics in Research Committee approval (processes 003/ 2012 and 560,994/2014) were received and all participants signed the Informed Consent Term.

### Study variables

Presence of cleft lip and palate was the dependent variable. Independent variables related to the child were gender, age, race/color of skin (according to the classification of the Brazilian Institute of Geography and Statistics) [ 22 ] and order of pregnancy. Independent variables related to parents were age and education level. Independent variables related to maternal exposure in the first trimester of pregnancy were occurrence of diabetes, hypertension, infection, obesity, use of medication (vitamin supplement, folic acid, analgesic, antibiotic, anti-inflammatory, corticosteroids, anticonvulsants and benzodiazepines), alcohol consumption, (active/ passive) smoking, illicit drug use, and exposure to ionizing radiation.

### Processing and data analysis

Data processing was carried out in an Excel spreadsheet and the statistical analyzes were performed with the Statistical Package for the Social Sciences (SPSS) version 17.0, MINITAB version 15.0 and STATA version 13.0. Tables presenting absolute and relative frequencies were used for the descriptive data analysis. In the inferential analysis, measures of association between dependent and independent variables were determined using Chi-square and Fisher's exact tests and Likelihood Ratio with a 0.05 significance level.

The crude odds ratio (OR) with their respective intervals of confidence of 95% (CI 95%) and the association between variables were also obtained, and those with *p*< 0.20 were considered for the construction of the multivariate logistic regression model, remaining in the final model the variables with significance level inferior than 0.05 (*p*< 0.05).

## Results

Regarding the socio-demographic data of the study population (n= 450), the predominance of male children (64.67%, n= 292), of white race/color of skin (43.56%, n = 196) was observed. A total of 63.78% (n= 287) of mothers aged from 20 to 34 years and 82.89% (n= 373) of fathers aged from 20 to 39 years. The level of education of 10 to 12 years of study prevailed in 63.11% (n= 284) of mothers and 50.67% (n= 228) of fathers. As for the data related to pregnancy, prenatal, occurrence of diseases, use of medication and social habits in the first trimester of pregnancy, it turns out that 97.78% (n= 440) of women received prenatal care and 40.22% (n= 181) were in first pregnancy. Only 2.44% (n=11) of mothers had diabetes, 7.33% (n=33) hypertension, 33.55% (n=151) infection, predominantly urinary and vaginal; 41.78% (n=188) psychological stress and 8.67% (n=39) were overweight at the beginning of pregnancy. Among medications use, the most frequently ones were folic acid (71.33%, n=321), vitamins (50.67%, n=228), analgesics (46.44%, n=209), antibiotics (25.33%, n=114) and anti-inflammatory (4.67%, n=21). Seventy- four women (16.22%) reported alcohol consumption, 1.56% (n=7) illicit drug use, 21.78% (n= 98) active or passive smoking and 2.44% (n=11) contact with ionizing radiation.

Tables 1 and 2 show the bivariate analysis of data, observing association (*p*< 0.05) between the following variables and the occurrence of oral clefts: Native-American race/color of skin (*p*<0.001RV), father education level ≤9 years (*p*=0.007), exposure to ionizing radiation (=0.008EF), passive smoking (*p*=0.010) and obesity (*p*=0.013). Being of black race/color of skin (*p*=0.006) and the use of analgesic (*p*<0.001) and folic acid (*p*<0.001) were associated with lower risk of occurrence of oral clefts.

**Table 1:** Association between sociodemographic variables and the occurrence of oral clefts.

Variable	Categories	Cases		Controls		OR	CI	*p* Value
		n	%	n	%		(95%)	(χ2)
Child gender	Male	97	33.33	194	66.67	1.00	(0.66 ; 1.51)	1.000
	Female	53	33.33	106	66.67	1.00	-	-
Child race/color	Native-American	5	100	0	0.00	-	-	0.001
	Black	24	20.17	95	79.83	0.48	(0.28 ; 0.81)	0.006
	Brown	53	40.77	77	59.23	1.30	(0.82 ; 2.05)	0.266
Father’s age at pregnancy	≤ 19 years	14	41.18	20	58.82	1.41	(0.69 ; 2.88)	0.350
	20 a 39 years	124	33.24	249	66.76	1.00	-	-
	≥ 40 years	12	27.91	31	72.09	0.78	(0.39 ; 1.57)	0.480
Mother’s age at pregnancy	≤ 19 years	46	35.11	85	64.89	1.11	(0.72 ; 1.72)	0.635
	20 a 34 years	94	32.75	193	67.25	1.00	-	-
	≥ 35 years	10	31.25	22	68.75	0.93	(0.42 ; 2.05)	0.863
Father’s education level	≤ 9 years	58	42.34	79	57.66	1.84	(1.18 ; 2.87)	0.007
	10 a 12 years	65	28.51	163	71.49	1.00	-	-
	> 12 years	19	39.58	29	60.42	1.64	(0.86 ; 3.14)	0.130
	Not informed	8	21.62	29	78.38	0.69	(0.30 ; 1.59)	0.384
Mother’s education level	≤9 years	45	38.79	71	61.21	1.41	(0.90 ; 2.21)	0.133
	10 a 12 years	88	30.99	196	69.01	1.00	-	-
	> 12 years	17	34.00	33	66.00	1.15	(0.61 ; 2.17)	0.672

OR: Odds Ratio; CI: confidence interval; p Value: Chi-square test.

**Table 2:** Association between pregnancy, prenatal, illnesses and life style variables and the occurrence of oral clefts

Variable	Categories	Cases		Controls		OR	CI	*p* Value
		n	%	n	%		(95%)	(χ2)
Order of pregnancy	First	62	34.25	119	65.75	0.99	(0.62 ; 1.58)	0.955
	Second	47	34.56	89	65.44	1.00	-	-
	Third	22	29.33	53	70.67	0.79	(0.43 ; 1.45)	0.439
	Fourth	9	23.08	30	76.92	0.57	(0.25 ; 1.30)	0.175
	Fifth or posterior	10	52.63	9	14.29	2.10	(0.80 ; 5.54)	0.126
Prenatal	Yes	144	32.73	296	67.27	1.00	-	-
	No	6	60.00	4	40.00	3.08	(0.86 ; 11.10)	0.091
Diabetes	Yes	5	45.45	6	54.55	1.69	(0.51 ; 5.63)	0.518EF
	No	145	33.03	294	66.97	1.00	-	-
Hypertension	Yes	16	48.48	17	51.51	1.99	(0.97 ; 4.06)	0.055
	No	134	32.13	283	67.87	1.00	-	-
Infection	Yes	56	37.09	95	62.91	1.29	(0.85 ; 1.94)	0.230
	No	94	31.44	205	68.56	1.00	-	-
Overweight	Grades 1 and 2	20	51.28	19	48.72	2.28	(1.17 ; 4.41)	0.013
	No	130	31.63	281	68.37	1.00	-	-
Stress	Yes	67	35.64	121	64.36	1.19	(0.80 ; 1.77)	0.380
	No	83	31.68	179	68.32	1.00	-	-
Use of folic acid	Yes	78	24.30	243	75.70	1.00	-	-
	No	72	55.81	57	44.19	3.94	(2.56 ; 6.06)	<0.001
Use of vitamins	Yes	82	35.96	146	64.04	1.00	-	-
	No	68	30.63	154	69.37	0.79	(0.53 ; 1.17)	0.230
Use of analgesics	Yes	47	22.49	162	77.51	0.39	(0.26 ; 0.59)	<0.001
	No	103	42.74	138	57.26	1.00	-	-
Use of antibiotics	Yes	46	40.35	68	59.65	1.51	(0.97 ; 2.34)	0.066
	No	104	30.95	232	69.05	1.00	-	-
Use of anti-inflamatory	Yes	7	33.33	14	66.67	1.00	(0.40 ; 2.53)	1.000
	No	143	33.33	286	66.67	1.00	-	-
Use of corticosteroids	Yes	1	25.00	3	3	0.66	(0.07 ; 6.44)	1.000EF
	No	149	33.41	297	297	1.00	-	-
Use of anticonvulsivants	Yes	4	66.67	2	2	4.08	(0.74 ; 22.54)	0.099EF
	No	146	32.88	298	298	1.00	-	-
Use of benzodiazepínes	Yes	4	66.67	2	33.33	4.08	(0.74 ; 22.54)	0.099EF
	No	146	32.88	298	67.12	1.00	-	-
Alcohol consumption	Yes	24	32.88	49	67.12	0.98	(0.57 ; 1.66)	0.928
	No	126	33.42	251	66.58	1.00	-	-
Frequency of alcohol consumption	1-7 times per week	23	37.10	39	62.90	1.18	(0.67 ; 2.05)	0.571
	1-2 times per month	1	9.09	10	90.91	0.20	(0.03 ; 1.57)	0.111EF
	0 times per month	126	33.42	251	66.58	1.00	-	-
Number of alcohol doses	≥ 5	7	43.75	9	56.25	1.55	(0.56 ; 4.26)	0.392
	1--4	17	29.82	40	70.18	0.85	(0.46 ; 1.55)	0.590
	None	126	33.42	251	66.58	1.00	-	-
Ilicit drug use	Yes	2	28.57	5	71.43	0.80	(0.15 ;4.16)	1.000EF
	No	148	33.41	295	66.59	1.00	-	-
Smoking	Active	13	46.43	15	53.57	2.04	(0.94 ; 4.43)	0.068
	Passive	32	45.71	38	54.29	1.98	(1.17 ; 3.34)	0.010
	No	105	29.83	247	70.17	1.00	-	-
Number of cigarettes per day	> 21	18	72.00	7	28.00	6.05	(2.45 ; 14.91)	<0.001
	11--20	12	40.00	18	60.00	1.57	(0.73 ; 3.37)	0.246
	< 10	15	34.88	28	65.12	1.26	(0.65 ; 2.46)	0.496
	No	105	29.83	247	70.17	1.00	-	-
X-ray	Yes	8	72.73	3	27.27	5.58	(1.46 ; 21.34)	0.008EF
	No	142	32.35	297	67.65	1.00	-	-

CI: confidence interval; p-value: Chi-square test. FE: Fisher's exact test.

No association was found with the other studied variables. The factors associated with the outcome after analysis on logistic regression multivariate model are presented in Table 3. The variables that remained as a risk factor for the occurrence of oral clefts by maternal exposure in the first trimester of pregnancy were: obesity (*p*= 0.001), passive smoking (*p*= 0.010) and exposure to ionizing radiation (*p*= 0.015). 

**Table 3:** Final model of logistic regression of the variables associated with oral clefts.

Variables/Categories	AOR	CI (95%)	*p* Value
Obesity			
Overweight grades 1 and 2	3.41	(1.61;7.26)	0.001
No	1.00	-	-
Use of folic acid			
Yes	1.00	-	-
No	4.17	(2.60;6.68)	<0.001
Use of analgesics			
Yes	0.35	(0.22;0.55)	<0.001
No	1.00	-	-
Use of antibiotics			
Yes	2.24	(1.35;3.72)	0.002
No	1.00	-	-
Smoking			
Active	1.53	(0.61;3.87)	0.366
Passive	2.18	21	0.010
No	1.00	-	-
X-ray			
Yes	6.95	(1.46;33.15)	0.015
No	1.00	-	-

AOR: adjusted *odds ratio*. CI (95%): confidence interval of 95%. *p* Values highlighted in bold are statistically significant (*p*< 0.05). Logarithm of likelihood value of the model= -246.9055 and p Value of the model <0.001.

The use of antibiotics was associated in the multivariate analysis (*p*= 0.002). The analgesic use was confirmed as a preventive factor (*p*< 0.001) as well as folic acid (*p*< 0.001).

## Discussion

Oral clefts are the oral malformations of higher incidence of in the world population [ 1 - 2 ], however the factors associated with its pathogenesis are still not completely defined [ 2 - 3 ]. Therefore, it is vital that new research be conducted with the purpose of helping unravel the etiology of this important public health issue [ 4 ].

Once gene therapy is still not available for the prevention of oral clefts [ 1 , 4 ] this study adhered to the investigation of maternal exposure to risk factors related to its occurrence within the first trimester of pregnancy. The identification of such factors would enable the establishment of preventive measures to prevent or control exposure by pregnant women, especially during the first trimester of pregnancy.

The decision of pairing the mothers of the case and control groups considering the gender of their children but not their age occurred in order to reduce the recall bias of the mothers from the control group. In births, where parents are faced with a very different child rather the idealized one, it naturally begins an internal process of finding a cause to explain the problem to minimize the feeling of guilt, so the pregnancy memories become more vivid. Baby without malformations and close to the idealized one become more difficult to be remembered over time [ 23 ]. Thus, the collection of data from the control group was conducted with the mothers still hospitalized in the postpartum.Among the sociodemographic variables analyzed in the present study, the Native-American race/color was associated with the occurrence of oral clefts (<0.001RV). Although similar results have been reported in studies conducted in other countries [ 13 - 14 ], it is possible that the incidence of congenital anomalies among Native-American be often underestimated due to the existence even nowadays of infanticide of malformed children [ 24 ]. In the present case, the association found may be related to the occurrence of consanguineous marriages, common in native communities [ 24 - 25 ]. However, the presence of Native-American individuals only in the case group should be considered with caution because it may represent an artificial result, not necessarily associated with a higher incidence of oral clefts in this population. While oral cleft natives search for treatment at the state capital referral service, native pregnant women hardly move to the capital to have their children, so that childbirth usually occurs in the own community [ 24 - 25 ] or in health institutions closer to the villages. Thus, the chance of Native-American being included in the control group is naturally less likely than in the case group.

Being of black race/color was considered in this study as a preventive factor to the oral clefts occurrence. Previous studies have observed a greater association between oral clefts and whites followed by brown [ 14 ], or brown followed by white [ 15 ], and a lower prevalence of all types of oral clefts among blacks compared to whites and Asians [ 13 - 14 , 26 ]. While studies have shown a significant relationship between the educational level of the mother and the risk of their occurrence [ 11 , 15 ]; others showed no interference between schooling and the occurrence of oral clefts [ 27 - 28 ]. 

This study showed an association between low paternal education and the occurrence of oral cleft (*p*= 0.007), unlike other studies that only analyzed the maternal level of education [ 11 , 27 - 28 ]. Whereas education is directly related to income [ 22 ], low schooling of the father may have hindered the pregnant women access to adequate nutrition, contributing to the occurrence of congenital malformation. 

Obesity has been identified as a risk factor for fetal malformations, such as neural tube defects, heart defects and orofacial clefts [ 16 ]. A study in Texas-EUA [ 17 ] found substantially increased risk of birth defects among obese mothers (BMI≥30), including cleft lip with or without cleft palate. Block *et al*. [ 18 ] found a relation between pregnancy obesity and ten birth defects, including isolated cleft palate. Stott-Miller *et al*. [ 28 ] also observed increased risk of isolated orofacial clefts among children of obese women.

In this study, the occurrence of cleft lip and palate was associated to obesity (*p*= 0.013), but not to diabetes (*p*= 0.518EF) and hypertension (*p*= 0.055), however the p Value was close to the statistical significance thresh old for the association of cleft lip and palate and the mother’s hypertension. The results showed that the incidence of births of children with oral clefts was strongly associated with non-folic acid supplementation by the mother in the first trimester of pregnancy (*p*< 0.001), representing an increase of 2.94 to 3.17 in the chance of oral cleft occurrence (3.94-1;4.17-1). The preventive effect of folic acid and vitamin supplements in the occurrence of cleft lip with or without cleft palate is a consensus [ 11 - 12 , 29 ]. In this study, the use of analgesics showed strong preventive association in the occurrence of oral cleft (*p*< 0.001). That can be attributed to the relaxing effect of the cessation of pain, as described in a previous study [ 30 ], which suggests that the physical and/or emotional stress may be implicated in the occurrence of oral clefts. The longer duration high tension can cause oxidative damage at cellular level by disruption of the hypothalamic-pituitary-adrenal axis leading to high levels of cortisol and cytokine production. This hormonal change leads to a decrease of the blood supply in the muscles, leading to decreased blood flow to the placenta causing a nutritional deficiency that can lead to genetic abnormalities in the fetus [ 30 ]. Studies have shown an association between the use of antibiotics, such as tetracycline, sulfamethoxa zole, trimethoprim, pivmecillinam [ 19 ] and amoxicillin [ 9 ] in early pregnancy and the risk of isolated orofacial clefts. Rocha *et al*. [ 8 ] found no statistical significance between the teratogenic risk for the use of antibiotics during pregnancy and the presence of fetal malformations. In the multivariate analysis results of this study, the use of antibiotics in early pregnancy was associated with the risk of oral clefts (*p*= 0.002) and deserves further investigation concerning the type and the prescribed dosage of antibiotic, given that the study was limited to investigating only the use or not use of the medication.

Smoking during pregnancy has often been associated with risk of oral clefts [ 6 , 9 ] regardless of race/color [ 5 ], because of reduced blood exchange between mother and fetus and fetal folate levels [ 9 ]. In this study, passive smoking of pregnant women was associated with the occurrence of oral clefts in their children (*p*= 0.010), observing even greater association in the group of children whose father smoked more than 21 cigarettes per day during the first trimester of pregnancy (*p*< 0.001).

The low number of pregnant smokers in this study may be related to the disclosure to the general population and during the prenatal consultations that smoking can induce the occurrence of birth defects. The association found between oral clefts and passive smoking, that is, the smoking habit of the partner, indicates that conducts aimed to reduce this habit from the partner and mother’s close people during pregnancy need to be disclosed as an important preventive measure for birth defects.

Another possibility is that the paternal smoking may exert some influence even before the pregnancy, interfering in the genesis of the male gamete that would end up generating a child with oral cleft [ 7 ]. This possibility to be confirmed requires further studies using different methodologies.

Although it was shown that the fetus is more susceptible to radiation between the second and fifteenth weeks of intrauterine life in a research on the risk of exposure to ionizing radiation resulting from medical procedures, Patel *et al*. [ 20 ] concluded that there is no acknowledged risk to the development of congenital malformation to fetuses exposed to ionizing radiation at levels typically used for diagnostic imaging. Rakotoarison *et al*. [ 21 ] pointed out the high doses of ionizing radiation from former uranium mines as a possible explanation for the high cleft prevalence in the Vakinankaratra region in Madagascar. In this study, exposure to ionizing radiation was associated with the presence of oral clefts (*p*= 0.015EF). The result, however, needs to be further explored, as the questionnaire limited the response to yes or no for questions whether mothers have been subjected to ionizing radiation in the first trimester of pregnancy or not. Thus, information on the frequency, body site and ionizing radiation levels to which they were submitted were not investigated, indicating a need for future studies. The findings of this study confirm the relationship between the occurrence of cleft lip and palate and behavioral maternal gestational conditions therefore likely to be prevented. Thus, the need for the monitoring of pregnant women is reiterated, especially in the first trimester of pregnancy, in order to limit or control their exposure to factors that were associated with its occurrence.

## Conclusion

Considering the methodology used in this study, it can be concluded that obesity, passive smoking, exposure to ionizing radiation and antibiotic use in the first trimester of pregnancy are associated with the occurrence of cleft lip and palate. The use of folic acid and analgesics and being of black race/color presented preventive effect for its occurrence. Thus, monitoring and careful controlling the identified risk factors in pregnant women, especially in the first trimester of pregnancy, is essential. 

Future studies are needed to clarify the relationship between risk factors and protective factors identified in this study and the occurrence of cleft lip and palate in the population.
